# Energy Allocation Resilience and Endocrine Integration

**DOI:** 10.3390/ijms27031345

**Published:** 2026-01-29

**Authors:** Corey B. Schuler, Allison B. Sayre, Lara Zakaria, Shawn Tassone, Alexander Rinehart, Richard Harris

**Affiliations:** 1 Department of Nursing, Augsburg University, Minneapolis, MN 55454, USA; 2 Independent Researcher, White Bear Lake, MN 55110, USA; 3 College of Nutrition, Sonoran University of Health Sciences, Tempe, AZ 85282, USA; 4 Department of Clinical Research and Leadership, Northwestern Health Sciences University, Bloomington, MN 55431, USA; 5 School of Medicine & Health Sciences, George Washington University, Washington, DC 20037, USA; 6 Independent Researcher, Austin, TX 78681, USA; 7 Independent Researcher, Scottsdale, AZ 85255, USA; 8 Independent Researcher, Houston, TX 77027, USA

**Keywords:** energy allocation, resilience, mitochondrial reserve capacity, hypothalamic-pituitary-adrenal axis, hypothalamic-pituitary-thyroid axis, hypothalamic-pituitary-gonadal axis, immunometabolism, stress physiology, thyroid hormone metabolism, energy governance

## Abstract

Resilience is commonly framed as a psychological trait, yet clinical and experimental evidence demonstrates that resilience failures emerge concurrently across metabolic, endocrine, immune, and cognitive domains. This review examines resilience as a bioenergetic property constrained by how organisms allocate finite metabolic resources under stress. We synthesize evidence from endocrinology, mitochondrial biology, immunometabolism, and stress physiology to propose a parsimonious, hypothesis-driven Energy Allocation System (EAS) in which the hypothalamic-pituitary-adrenal (HPA), thyroid (HPT), and gonadal (HPG) axes are conceptualized as a coordinated energy-governance network. Despite extensive investigation within these individual fields, the literature lacks an integrative physiological framework explaining why multisystem stress responses co-occur in predictable endocrine and metabolic patterns. Within this framework, mitochondrial reserve capacity serves as the limiting substrate through which hormonal signals regulate mobilization, metabolic pacing, immune tolerance, and recovery. The reviewed literature supports predictable patterns of endocrine reorganization during energetic strain, including prioritization of glucocorticoid-mediated mobilization, constrained thyroid hormone activation, suppression of long-term anabolic investment, and impaired recovery following stress. These configurations reflect adaptive energy-conserving strategies rather than isolated organ dysfunction. The novelty of this review lies in organizing established biological mechanisms into a unified, energy-allocation-based framework that generates falsifiable predictions linking endocrine coordination to bioenergetic capacity and recovery dynamics. We further discuss how routinely available biomarkers and validated psychometric measures can be interpreted as functional readouts of energetic allocation rather than static disease markers. Framing resilience through coordinated energy governance offers a unifying mechanistic lens for interpreting multisystem stress responses and generates testable predictions for future experimental and clinical investigation.

## 1. Introduction

Resilience, the capacity to maintain or restore function under physiological or psychological stress, is increasingly recognized as a multisystem biological phenomenon rather than a purely psychological trait [1]. Clinical and experimental observations consistently demonstrate that resilience failures manifest simultaneously across metabolic, endocrine, immune, and cognitive domains, producing recurring constellations of fatigue, mood disturbance, metabolic inflexibility, immune dysregulation, and impaired recovery following stressors [2,3]. Despite extensive investigation within individual disciplines, a unifying physiological framework explaining why these systems fail together, and in predictable patterns, remains incomplete.

Stress biology has traditionally focused on hypothalamic-pituitary-adrenal (HPA) axis activation, glucocorticoid signaling, and downstream inflammatory consequences [3]. In parallel, endocrinology has characterized stress-associated alterations in thyroid and gonadal axes [4] while mitochondrial biology has established that cellular adaptive capacity is constrained by oxidative phosphorylation efficiency and reserve respiratory capacity [5]. This literature, however, is often treated as mechanistically adjacent rather than functionally integrated. As a result, common clinical presentations, such as stress-associated reductions in thyroid hormone activation despite normal central drive, suppression of reproductive signaling during chronic stress, or immune polarization toward tolerance states [4,6], are frequently interpreted as isolated dysfunctions rather than coordinated adaptive responses [7]. Central to this framework is the recognition that the physiological consequences of stress are determined not solely by the magnitude of stress exposure, but by the duration and persistence of energetic demand, which shape whether adaptive responses remain reversible or consolidate into longer-term allocation states.

Here, we propose an integrative, hypothesis-driven heuristic framework that does not introduce new biological mechanisms, but instead organizes established stress, endocrine, immune, and metabolic processes around the principle of energy allocation across timescales.

A growing body of evidence suggests that energy availability represents a shared constraint linking these phenomena. In this framework, “shared bioenergetic constraint” refers not to direct sharing of adenosine triphosphate (ATP) or mitochondrial machinery between tissues, but to organism-level limitations in fuel availability, oxygen delivery, redox balance, and perfusion-dependent recovery capacity that shape how local mitochondrial function is supported across systems. Mitochondrial energy production underpins endocrine signaling, immune activation, neural computation, and tissue repair, yet ATP generation is finite and dynamically regulated [5]. Immune activation, oxidative stress management, and sustained neuroendocrine mobilization impose substantial energetic costs, requiring organisms to prioritize resource allocation among competing physiological demands [8]. From an evolutionary perspective, such prioritization favors short-term survival under threat at the expense of long-term investment in growth, reproduction, and restoration.

Within this context, endocrine axes can be viewed as components of an integrated energy-allocation network. The HPA axis regulates rapid substrate mobilization [2]; the hypothalamic-pituitary-thyroid (HPT) axis modulates mitochondrial oxidative capacity and metabolic pace [9]; and the hypothalamic-pituitary-gonadal (HPG) axis supports energetically expensive anabolic, reproductive, and immune tolerance functions [10]. Rather than operating independently, we propose these axes interact through shared dependence on mitochondrial reserve capacity and redox state, coordinating how energy is mobilized, converted, invested, and restored under varying conditions of stress and recovery.

The aim of this review is to synthesize evidence from endocrinology, mitochondrial bioenergetics, immunometabolism, and stress physiology to propose an integrated Energy Allocation System (EAS) framework for understanding resilience. By framing resilience as an emergent property of coordinated energy governance across endocrine axes, this model offers a conceptual lens for interpreting multisystem stress responses and generates testable predictions regarding endocrine patterns, metabolic constraints, and recovery dynamics observed in both experimental and clinical settings. Because all adaptive responses require metabolic energy, an examination of bioenergetic constraints provides a necessary foundation for understanding coordinated endocrine regulation under stress.

## 2. Bioenergetic Constraints on Stress Adaptation

Here, we conceptualize physiological resilience as fundamentally constrained by the capacity of biological systems to generate, distribute, and restore metabolic energy under stress across time. Across molecular, cellular, and organismal levels, adaptive responses depend on the ability to increase ATP production in proportion to demand while maintaining redox balance and cellular integrity. When energetic capacity is exceeded, systems must prioritize immediate survival over long-term investment, producing predictable trade-offs across endocrine, immune, and cognitive domains.

### 2.1. Mitochondrial Reserve Capacity as a Limiting Substrate

Mitochondrial reserve capacity, also referred to as spare respiratory capacity, describes the ability of mitochondria to increase ATP production above basal requirements in response to acute or sustained stress. This reserve represents the energetic margin that allows cells and tissues to adapt to increased workload without loss of function. Reduced reserve capacity predicts heightened vulnerability to oxidative stress, impaired cellular survival, and diminished adaptive flexibility [5].

Within the context of stress adaptation, mitochondrial reserve capacity functions as a system-wide constraint rather than a purely cellular property [11]. When reserve capacity is sufficient, endocrine signaling, immune activation, and neural computation can proceed in parallel without excessive trade-offs [12]. When reserve capacity is limited, whether by inflammation, insulin resistance, micronutrient deficiency, circadian disruption, or cumulative stress exposure, adaptive responses compete for ATP, forcing prioritization decisions that favor short-term mobilization over restoration and long-term investment [5,7,11,13].

Bioenergetic reserves may be constrained by micronutrient availability and physical activity, both of which are modifiable. Iron, selenium, iodine, folate, and vitamins D (cholecalciferol) and B12 (cobalamin) are required for mitochondrial electron transport, thyroid hormone synthesis and conversion, antioxidant defense, and immune regulation [14,15,16,17,18,19]. Deficiencies in these substrates plausibly compress energetic margins independent of primary endocrine disease, increasing the ATP cost of adaptation under stress. Physical activity induces systemic molecular and cellular adaptations, including enhanced mitochondrial function and energy homeostasis, that support resilience by improving the body’s capacity to recover, adapt, and thrive in response to physiological and psychosocial stressors [20]. Theoretically, insufficiency in physical activity may constrain adaptive energy, potentially via mitochondrial reserve capacity.

Experimental models demonstrate that stress response magnitude alone is a poor predictor of allostatic burden; instead, outcomes depend on the organism’s capacity to meet energetic demand during stress exposure [11]. Individuals or animal models with preserved mitochondrial function maintain adaptive responses despite similar stress loads, whereas those with compromised bioenergetics exhibit exaggerated fatigue, cognitive impairment, metabolic dysregulation, and delayed recovery [2,12].

Within this framework, hormesis provides additional context for understanding how mitochondrial reserve capacity may be dynamically expanded or eroded in response to stress exposure. Hormesis describes a biphasic dose–response relationship in which low-intensity or transient stressors, including hormetins, promote adaptive biological responses, whereas excessive or sustained exposure becomes maladaptive [21,22,23]. Related mitohormetic processes position mitochondria as central signaling hubs, whereby modest energetic or redox challenges activate adaptive pathways involved in antioxidant defense, mitochondrial maintenance, and metabolic flexibility [24]. These responses are coordinated in part through transcriptional regulators such as nuclear factor erythroid 2-related factor 2 (Nrf2), which integrates mitochondrial redox signaling with broader cellular stress responses [25,26]. Importantly, mitohormetic signaling interfaces with endocrine regulation, including the hypothalamic-pituitary-adrenal (HPA), hypothalamic-pituitary-thyroid (HPT), and hypothalamic-pituitary-gonadal (HPG) axes, linking mitochondrial adaptation to system-level energy allocation decisions [22,23]. Consistent with the Energy Allocation System (EAS), the adaptive or maladaptive direction of these responses remains fundamentally dose- and context-dependent, constrained by the organism’s available energetic reserve [21]. From a translational standpoint, this dose-sensitive hormetic framework may inform future hypothesis-driven therapeutic investigations by clarifying the boundary conditions under which functional nutrients or pharmacologic agents acting as hormetins engage Nrf2 signaling and neuroendocrine axes in relation to metabolic health and stress resilience, while minimizing the risk of maladaptive overactivation.

### 2.2. Energetic Cost of Immune Activation and Inflammatory Load

Immune activation imposes a substantial energetic cost [13]. Both innate and adaptive immune responses require rapid ATP generation to support cellular proliferation, cytokine synthesis, reactive oxygen species handling, and tissue surveillance. Pro-inflammatory states therefore act as energetic sinks, diverting ATP away from endocrine signaling, mitochondrial repair, and cognitive processing [8,13].

Low-grade chronic inflammation has been shown to impair mitochondrial substrate switching, reduce oxidative phosphorylation efficiency, and increase reliance on glycolytic metabolism [8]. Cytokines such as interleukin-6 (IL-6) and tumor necrosis factor-α (TNF-α) directly modulate mitochondrial function, increase oxidative stress, and alter insulin signaling, further compressing the energetic margin available for adaptive responses [27]. Immune activation itself is energetically demanding. Even modest increases in IL-6 or C-reactive protein (CRP) correspond to elevated ATP demand for cellular defense, mitochondrial ROS management, and tissue surveillance, which tends to redirect resources away from high-cost endocrine and cognitive processes, potentially contributing to fatigue and impaired recovery under stress [28,29].

From an energetic perspective, immune polarization toward tolerance-oriented phenotypes reflects a cost-containment strategy. Th2 and regulatory T-cell (Treg) responses operate at lower metabolic cost than cytotoxic Th1-dominant states, conserving ATP under conditions of sustained stress [6,30]. This shift is frequently observed in chronic stress models and inflammatory states, reinforcing the concept that immune configuration reflects energetic availability rather than isolated immune dysfunction [31]. These immunometabolic demands interact closely with endocrine signaling pathways, which are examined in subsequent sections as regulators of energy allocation under sustained stress.

### 2.3. Stress-Induced Endocrine Reorganization

Stress exposure increases energetic demand across multiple physiological systems simultaneously. Activation of the HPA axis promotes rapid substrate mobilization but also elevates metabolic cost through sustained glucocorticoid signaling, increased gluconeogenesis, and inflammatory modulation [2]. Neural stress responses further increase ATP demand through heightened synaptic activity, neurotransmitter turnover, and network-level coordination [12,32]. In this context, persistent configurations are understood as adaptive, energy-conserving responses to sustained demand, rather than as intrinsic pathological failures, becoming maladaptive only when energetic constraints remain unresolved.

When energetic demand persists, these costs accumulate. Chronic stress has been associated with impaired insulin sensitivity, reduced mitochondrial efficiency, altered circadian signaling, and delayed recovery following perturbation [3]. These effects are not independent but synergistic: metabolic inflexibility increases reliance on stress-mediated mobilization, inflammation impairs mitochondrial throughput, and circadian disruption limits nighttime restoration of energetic reserves [33,34,35].

From an evolutionary standpoint, such prioritization reflects conserved survival logic [13]. Under conditions of energetic scarcity, organisms favor immediate threat readiness at the expense of energetically expensive processes such as reproduction, anabolic repair, and long-term immune defense. This reallocation preserves short-term survival but reduces resilience capacity over time when energetic constraints are not resolved [36]. Within the EAS framework, diurnal patterns become substantially relevant, given the consideration of time scales. As an example, the cortisol awakening response (CAR) can be interpreted as a marker of temporal energy allocation efficiency, reflecting appropriate mobilization during waking hours and restoration during the night. Flattened or blunted CAR patterns may therefore indicate impaired energetic scheduling rather than isolated dysregulation of HPA output [37].

### 2.4. Path-Dependent Consequences for Multisystem Resilience

From an energetic perspective, fatigue, cognitive slowing, reduced motivation, and impaired working memory represent functional manifestations of constrained ATP availability rather than nonspecific or secondary symptoms. Neuroimaging and behavioral studies in stress-related exhaustion and chronic occupational stress demonstrate that these functional changes co-vary with inflammatory load and metabolic strain, consistent with an energy-budget model in which increasing allocation toward stress mobilization and immune regulation limits resources available for prefrontal function, mitochondrial repair, and recovery [38,39,40,41,42].

Taken together, these findings support the view that resilience is constrained not by stress exposure alone but by the energetic context in which stress occurs. While mitochondrial reserve capacity defines the upper limit of adaptive responding, immune activation and metabolic inefficiency erode the available energetic margin. When ATP demand persistently exceeds supply, systems reorganize predictably, prioritizing mobilization and conservation over restoration [2,5,11].

These bioenergetic constraints provide the mechanistic substrate upon which endocrine axes coordinate adaptive responses. The following section examines how the hypothalamic-pituitary-adrenal, thyroid, and gonadal axes function as regulators of energy allocation under these conditions, translating bioenergetic limitation into system-level physiological patterns.

## 3. Endocrine Axes as Regulators of Energy Allocation

While bioenergetic capacity defines the upper limit of adaptive responding, endocrine signaling governs how available energy is mobilized, distributed, and conserved across physiological systems [7]. The HPA, HPT, and HPG axes function as interdependent regulators of energy allocation, translating environmental demands and internal energetic status into coordinated physiological responses [34]. Rather than operating independently, these axes share a common dependence on mitochondrial ATP availability and redox balance, enabling dynamic prioritization among competing processes such as stress responsiveness, metabolic pacing, immune activity, and long-term anabolic investment [7,11]. Within this framework, endocrine outputs can be understood as adaptive energy-allocation signals constrained by underlying bioenergetic capacity. Although these axes operate on distinct timescales, none function hierarchically; instead, their relative influence reflects shared energetic constraints and contextual demand.

### 3.1. HPA Axis: Energetic Mobilization Under Stress

The HPA axis serves as the primary mechanism for rapid energy mobilization in response to perceived threat. Throughout this review, “energy mobilization” refers to hormonally and neurally mediated increases in substrate release, tissue perfusion, and mitochondrial respiratory throughput that raise local ATP production capacity in response to demand. Mechanistically, activation of corticotropin-releasing hormone (CRH) neurons and subsequent glucocorticoid secretion promote immediate substrate availability through increased gluconeogenesis, lipolysis, and proteolysis, supporting acute cognitive, cardiovascular, and immune demands [2]. From an energetic perspective, HPA activation represents a high-priority allocation strategy that favors short-term survival over long-term physiological investment [43].

Glucocorticoid signaling exerts widespread metabolic effects that extend beyond substrate mobilization. Sustained HPA activation increases energetic cost through enhanced hepatic glucose output, altered insulin sensitivity, and modulation of mitochondrial function, including increased reactive oxygen species production and impaired oxidative phosphorylation efficiency under chronic exposure [3]. These effects amplify ATP demand while simultaneously constraining mitochondrial reserve capacity, narrowing the energetic margin available for recovery and repair [5].

Importantly, the magnitude of HPA activation alone does not determine adaptive outcome [2]. Experimental and clinical observations indicate that individuals with preserved bioenergetic capacity tolerate repeated or prolonged HPA activation with minimal functional decline, whereas those with compromised mitochondrial function exhibit exaggerated fatigue, cognitive impairment, and delayed recovery despite comparable glucocorticoid exposure [11,12]. This dissociation underscores the role of energetic context in shaping stress resilience.

Chronic reliance on HPA-mediated mobilization further influences downstream endocrine axes. Elevated glucocorticoids suppress gonadotropin-releasing hormone (GnRH) signaling and alter thyroid hormone metabolism, reinforcing an energy-conserving state that deprioritizes reproduction, anabolic repair, and long-term immune defense during sustained stress exposure [2,7]. These interactions position the HPA axis not as an isolated stress pathway, but as a dominant driver of system-wide energy reallocation under conditions of energetic strain.

### 3.2. HPT Axis: Metabolic Pacing and Mitochondrial Throughput

Emerging evidence suggests that reverse triiodothyronine (rT3), traditionally regarded as an inactive by-product of thyroid hormone metabolism, may actively inhibit T3 signaling at the tissue level. Elevated rT3, commonly observed during stress or illness, can reduce effective thyroid hormone bioavailability and contribute to a state of functional hypothyroidism despite normal TSH concentrations. These patterns have been associated with mood, cognitive, and behavioral dysfunction, supporting rT3 as a potentially informative biomarker of thyroid-mediated energy conservation under stress [44].

Whereas the HPA axis governs rapid energy mobilization, the HPT axis regulates the pace at which energy is produced and utilized across tissues [9]. Thyroid hormones modulate mitochondrial biogenesis, oxidative phosphorylation efficiency, and basal metabolic rate, positioning the HPT axis as a central regulator of long-term energetic throughput rather than acute substrate availability [45]. Through these actions, thyroid signaling determines whether available metabolic resources are directed toward sustained work capacity, repair, and recovery or conserved under conditions of energetic strain.

At the cellular level, triiodothyronine (T3) enhances mitochondrial respiration, increases expression of oxidative enzymes, and promotes coupling efficiency within the electron transport chain [11,46]. These effects increase ATP-generating capacity but also elevate energetic demand and oxidative burden. Consequently, thyroid hormone signaling must remain tightly coupled to mitochondrial reserve capacity and redox state to preserve adaptive flexibility. Excessive thyroid-driven throughput in the context of limited energetic reserve increases vulnerability to oxidative stress and functional exhaustion, whereas constrained thyroid signaling can serve as an energy-conserving adaptation [9,12].

Stress exposure alters thyroid hormone metabolism in predictable ways [9]. Chronic activation of the HPA axis suppresses peripheral conversion of thyroxine (T4) to T3 while increasing production of reverse T3 (rT3), effectively reducing mitochondrial stimulation without necessarily altering central thyroid-stimulating hormone (TSH) drive [7,45]. This pattern reflects a decoupling of central endocrine signaling from peripheral energetic execution, enabling conservation of ATP during sustained stress or inflammatory load. Such changes are frequently observed in stress-related mood disorders, chronic illness, and inflammatory states, even in the absence of overt thyroid disease [4].

From an energy-allocation perspective, reduced T3 availability represents a strategic downshifting of metabolic pace rather than primary glandular failure [7,9]. By limiting mitochondrial throughput, the organism preserves residual reserve capacity for essential functions while minimizing oxidative damage and energetic overextension. However, prolonged suppression of thyroid-driven metabolism also constrains tissue repair, cognitive performance, and immune competence, contributing to the symptom clusters commonly attributed to “functional hypothyroidism” despite TSH values within accepted reference ranges often termed biochemically euthyroid [45].

Importantly, thyroid signaling interacts bidirectionally with immune and metabolic pathways. Pro-inflammatory cytokines impair deiodinase activity, reduce thyroid hormone receptor sensitivity, and alter mitochondrial substrate utilization, reinforcing a low-throughput energetic state under inflammatory stress [8,47]. Insulin resistance and circadian disruption further exacerbate these effects, limiting the capacity for nocturnal restoration and compounding energetic constraint [27,29,48,49]. Within this framework, alterations in thyroid hormone metabolism function as adaptive responses to energetic limitation but become maladaptive when stressors persist without recovery [47].

Taken together, the HPT axis operates as a metabolic governor that aligns mitochondrial throughput with available energetic reserve [7]. In the context of chronic stress or inflammation, adaptive suppression of thyroid signaling conserves ATP and limits oxidative burden but simultaneously reduces resilience capacity over time [9,12]. These dynamics position the HPT axis as a critical mediator between bioenergetic constraint and system-wide functional output, linking cellular metabolism to organism-level adaptability.

### 3.3. HPG Axis: Long-Term Investment and Immune Tolerance

The HPG axis regulates energetically expensive processes associated with long-term biological investment, including reproduction, tissue anabolism, and immune modulation [13]. Gonadal steroids such as estrogen, progesterone, and testosterone exert widespread effects on mitochondrial function, immune cell metabolism, and inflammatory signaling, positioning the HPG axis as a key determinant of whether energy is allocated toward growth, repair, and tolerance rather than immediate threat responsiveness [10,50,51]. Within an energy allocation framework, robust HPG signaling reflects energetic sufficiency, whereas suppression of this axis signals prioritization of short-term survival over long-term investment.

Stress exposure reliably suppresses HPG activity through multiple mechanisms. Elevated glucocorticoids inhibit hypothalamic gonadotropin-releasing hormone (GnRH) pulsatility, reduce pituitary luteinizing hormone (LH) and follicle-stimulating hormone (FSH) secretion, and impair gonadal steroidogenesis [2,50,51]. Inflammatory cytokines further disrupt gonadal signaling by directly inhibiting steroidogenic enzymes and altering mitochondrial function within gonadal tissues, compounding the suppressive effects of chronic stress and metabolic strain [11,34]. These changes are frequently observed across diverse stress-related conditions, including chronic illness, undernutrition, and prolonged psychological stress [52,53]. Methylation is a central mechanism through which the organism encodes long-term energetic priorities. DNA methylation of the glucocorticoid receptor gene *NR3C1* reduces receptor expression and weakens HPA negative feedback, leading to prolonged cortisol exposure and elevated mobilization cost under stress [3,4]. Methylation patterns across NR3C1 also predict cardiovascular and endocrine reactivity, indicating that early epigenetic signals set energetic “baselines” that persist across the lifespan [54]. However, methylation patterns modulate rather than fully determine endocrine behavior, shaping tendencies in axis responsiveness without rigidly fixing physiological outcomes.

Within the EAS framework, these methylation programs establish long-standing “rules of engagement” governing how the HPA, HPT, and HPG axes allocate energy under threat, scarcity, or metabolic strain. They shape not only what an axis does under stress, but how much energy the organism is “willing to spend” to maintain mobilization, metabolism, or reproduction. As such, methylation patterns become measurable contributors to an individual’s resilience phenotype.

Beyond reproductive effects, gonadal steroids play a critical role in shaping immune phenotypes. Estrogens and androgens modulate immune cell differentiation, cytokine production, and metabolic programming, influencing the balance between pro-inflammatory and tolerance-oriented immune responses [8,10,50]. Adequate HPG signaling supports regulatory and reparative immune states that favor tissue maintenance and long-term resilience, whereas suppression of gonadal steroids shifts immune function toward energy-conserving configurations under stress [50].

From a metabolic perspective, immune tolerance represents a lower-energy operational state. Regulatory T cells and Th2-dominant responses require less ATP and generate lower oxidative burden than cytotoxic Th1-driven immunity, aligning immune function with constrained energetic conditions [6,55]. Suppression of HPG signaling during stress therefore reinforces a coordinated energy-conservation strategy, limiting the energetic cost of immune activation while preserving essential defensive capacity.

However, prolonged HPG suppression carries functional trade-offs. Reduced gonadal steroid availability compromises muscle mass maintenance, bone density, neuroplasticity, and vascular health, while sustained immune tolerance may impair pathogen clearance and tissue regeneration [28,51,56]. These consequences highlight the dual role of HPG suppression as both an adaptive response to energetic limitation and a contributor to resilience decline when stressors persist without restoration.

Within the Energy Allocation System framework, the HPG axis functions as a sentinel of energetic sufficiency. Its suppression signals a shift away from long-term investment toward conservation, completing the coordinated endocrine response to sustained energetic strain. Together with the HPA and HPT axes, HPG signaling integrates bioenergetic status with immune and reproductive priorities, shaping the organism’s capacity for resilience across time [7].

### 3.4. Sympathetic Nervous System: Fast-Timescale Neural Regulation of Energy Allocation

In addition to endocrine regulation, rapid neural signaling via the sympathetic nervous system (SNS) provides a fast-acting mechanism for immediate energy allocation [11,57]. Sympathetic activation rapidly increases cardiac output, substrate mobilization, and skeletal muscle perfusion, enabling near-instantaneous redistribution of energetic resources in response to perceived threat or demand [57,58].

**Figure 1:**
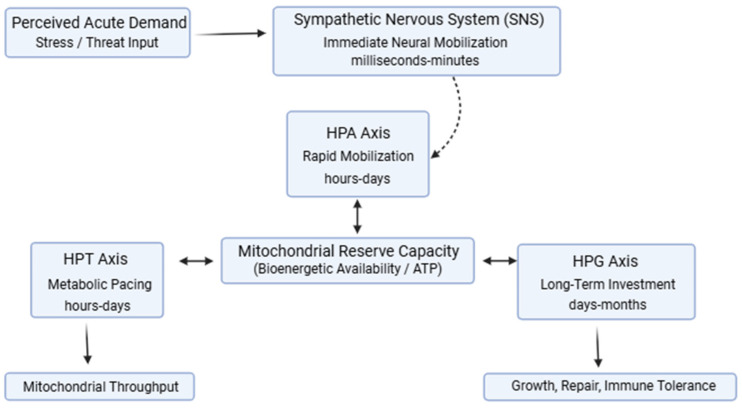
Conceptual schematic of the Energy Allocation System (EAS). Perceived acute demand engages rapid neural signaling via the sympathetic nervous system (SNS), providing fast-timescale mobilization of energetic resources. Endocrine axes regulate energy allocation across longer temporal horizons: the HPA axis supports rapid mobilization, the HPT axis modulates metabolic pacing and mitochondrial throughput, and the HPG axis governs long-term investment in growth, repair, and immune tolerance. All regulatory signals operate under constraints imposed by mitochondrial reserve capacity, which defines available bioenergetic supply. Neural and endocrine mechanisms act in parallel rather than in hierarchy. Figure created in https://BioRender.com.

Within the Energy Allocation System (EAS), the SNS functions as a parallel governor operating on sub-second to minute timescales, whereas endocrine axes regulate energy allocation over longer temporal horizons [43]. Neural and endocrine mechanisms therefore act in concert rather than in hierarchy: sympathetic signaling initiates immediate mobilization, while sustained allocation patterns are shaped by coordinated HPA, HPT, and HPG axes responses constrained by mitochondrial reserve capacity, as illustrated in Figure 1.

This temporal complementarity explains why acute sympathetic activation can occur without sustained endocrine reorganization, whereas chronic stress exposure requires longer-term endocrine recalibration to manage cumulative energetic cost.

## 4. An Integrated Energy Allocation System (EAS)

The preceding sections suggest that bioenergetic capacity constrains adaptive potential and that endocrine axes regulate how limited energy resources are mobilized, paced, and conserved under stress. Taken together, these findings support the existence of an integrated Energy Allocation System (EAS) in which mitochondrial ATP production capacity provides the limiting substrate, and coordinated endocrine signaling governs energy distribution across physiological domains. Within this framework, resilience emerges not from the isolated performance of individual systems but from the coherence with which energy is functionally shared, prioritized, and reallocated across timescales. Here, energy “sharing” refers to the regulated distribution of metabolic substrates (glucose, fatty acids, amino acids), oxygen, blood flow, and hormonal signaling priority across tissues, rather than direct transfer of ATP between cells.

The EAS conceptualizes the hypothalamic–pituitary–adrenal (HPA), thyroid (HPT), and gonadal (HPG) axes as functionally interdependent regulators operating on distinct energetic horizons. The HPA axis enables rapid mobilization of substrates to meet acute demands, the HPT axis modulates mitochondrial throughput and metabolic pace over intermediate timescales, and the HPG axis supports energetically costly processes associated with long-term investment, including reproduction, tissue repair, and immune tolerance. These axes do not act independently; rather, they converge on shared bioenergetic constraints defined by mitochondrial reserve capacity, redox balance, and substrate availability [5,11].

Under conditions of energetic sufficiency, coordinated endocrine signaling permits simultaneous investment in stress responsiveness, metabolic maintenance, immune competence, and restoration. Mitochondrial reserve capacity buffers transient increases in demand, allowing systems to flex without requiring trade-offs. In this state, endocrine signals remain proportionate to energetic capacity, supporting resilience and rapid recovery following perturbation. However, when energetic demand persistently exceeds supply, whether due to chronic stress, inflammation, metabolic dysfunction, or circadian disruption, adaptive responses must be re-prioritized. The integrated Energy Allocation System, illustrating coordinated regulation of mobilization, metabolic pacing, and long-term investment by the HPA, HPT, and HPG axes under bioenergetic constraint, is summarized schematically in Figure 1.

Within the EAS, we propose that sustained energetic strain drives predictable shifts in endocrine configuration. Prolonged HPA activation dominates energy allocation toward mobilization, while peripheral thyroid hormone activation is constrained to reduce mitochondrial throughput and conserve ATP [7]. Concurrent suppression of HPG signaling deprioritizes anabolic investment and reinforces immune tolerance, reducing energetic expenditure associated with growth, reproduction, and high-cost immune responses [10,51]. These coordinated adjustments reflect adaptive energy-conserving strategies rather than isolated endocrine failures.

Importantly, the EAS does not imply linear causality or hierarchical dominance among endocrine axes [7]. Instead, it describes a dynamic, constraint-based system in which signaling outputs are modulated by energetic context. Endocrine signals retain their regulatory intent, but their physiological effects are scaled according to mitochondrial capacity and competing energetic demands [11]. This framework provides a parsimonious interpretive model for understanding why similar hormonal patterns may produce divergent functional outcomes across individuals, depending on underlying bioenergetic reserve and inflammatory load [59,60].

By integrating mitochondrial bioenergetics with endocrine regulation, the EAS provides a conceptual bridge between molecular energy metabolism and organism-level resilience. It is consistent with the interpretation that the frequent co-occurrence of endocrine alterations, immune reconfiguration, and metabolic inflexibility under chronic stress [34], and offers a framework for understanding why interventions targeting single hormones or pathways often yield incomplete or transient benefits when energetic constraints remain unaddressed.

The EAS framework is intended as a synthesis of established biological principles rather than a replacement for existing models of stress adaptation. It complements concepts such as allostatic load by specifying the energetic mechanisms through which adaptive costs accumulate and resilience erodes over time [2,61]. By foregrounding energy allocation as the organizing principle, this model generates testable predictions regarding endocrine patterns, recovery dynamics, and vulnerability to multisystem dysfunction under conditions of sustained energetic strain.

## 5. Predictable Energetic Configurations and Testable Implications

**Figure 2:**
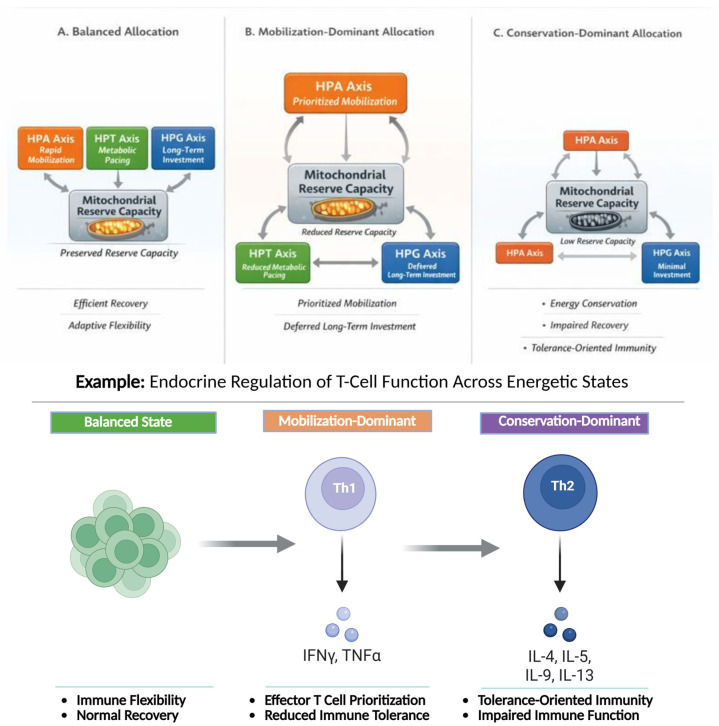
Predictable energetic configurations within the Energy Allocation System (EAS). Distinct endocrine patterns emerge as bioenergetic reserve capacity declines, reflecting adaptive prioritization among rapid mobilization (HPA axis), metabolic pacing (HPT axis), and long-term investment and immune tolerance (HPG axis). These configurations represent dynamic, reversible states shaped by energetic availability rather than fixed pathological entities. Inset: A representative exemplar illustrates how coordinated endocrine signaling shapes a specific target function of T-cell activation versus tolerance across balanced, mobilization-dominant, and conservation-dominant energetic states, linking system-level energy allocation to tissue-level functional outcomes across time scales. Figure created with BioRender.com.

The Energy Allocation System (EAS) predicts that sustained energetic constraints give rise to recurrent, system-level configurations characterized by coordinated changes in endocrine signaling, immune behavior, and metabolic throughput [11,34]. These configurations are not discrete disease entities but dynamic, potentially reversible states that reflect how organisms prioritize energy use under varying conditions of demand and recovery capacity. As illustrated in Figure 2, progressive limitation of mitochondrial reserve capacity produces predictable shifts in endocrine axis engagement across the HPA, HPT, and HPG systems. These configurations are not diagnostic categories but adaptive, reversible states reflecting how energy is prioritized under constraint [2,13].

### 5.1. Balanced Allocation Under Energetic Sufficiency

Under conditions of adequate bioenergetic reserve, endocrine signaling remains proportionate to demand, allowing coordinated engagement of stress responsiveness, metabolic pacing, and long-term investment. HPA activation is transient and appropriately scaled, thyroid hormone signaling supports efficient mitochondrial throughput, and gonadal steroid activity sustains anabolic processes and immune tolerance. In this configuration, mitochondrial reserve capacity buffers fluctuations in demand, enabling rapid recovery following stress exposure without necessitating prolonged trade-offs among systems.

This balanced state supports adaptive flexibility across physiological domains. Immune responses remain effective without excessive energetic cost, cognitive and physical performance are preserved, and restorative processes proceed during periods of reduced demand. Importantly, this configuration does not imply the absence of stress but reflects sufficient energetic capacity to accommodate stress without persistent reallocation.

### 5.2. Mobilization-Dominant Allocation During Compensated Stress

When energetic demand increases or reserve capacity begins to decline, the EAS predicts a shift toward mobilization-dominant allocation. In this configuration, HPA-mediated energy mobilization is prioritized to meet immediate demands [2], while thyroid-driven metabolic throughput is constrained and long-term investment through the HPG axis is deferred. This pattern is adaptive in the short term, enabling continued performance under elevated workload, illness, or psychosocial stress.

Physiologically, this state is characterized by increased reliance on glucocorticoid-mediated substrate mobilization, altered peripheral thyroid hormone conversion favoring reduced mitochondrial stimulation, and early suppression of gonadal signaling [2,7]. Although functional capacity may be maintained, recovery becomes increasingly dependent on stress resolution and restoration of energetic reserves. Prolonged residence in this configuration increases vulnerability to fatigue, cognitive strain, and metabolic inflexibility as energetic costs accumulate [39,53].

### 5.3. Conservation-Dominant Allocation Under Sustained Energetic Strain

With persistent stress exposure, inflammation, or impaired recovery, mitochondrial reserve capacity may become sufficiently constrained to necessitate broad energy conservation [5]. In this configuration, metabolic pacing and long-term investment are markedly suppressed, while immune function shifts toward tolerance-oriented, lower-energy phenotypes [6]. HPA activity may remain elevated or exhibit reduced variability, reflecting ongoing energetic strain without effective resolution.

This conservation-dominant state represents a protective adaptation that minimizes further energetic depletion and oxidative damage. Reduced thyroid hormone activity limits mitochondrial throughput [46], while suppression of gonadal signaling conserves resources otherwise allocated to reproduction, tissue growth, and high-cost immune activation [10]. However, prolonged persistence in this configuration constrains resilience capacity [36], impairing tissue repair, immune responsiveness, and functional recovery over time.

### 5.4. Testable Implications and Observational Readouts

To facilitate empirical evaluation, the Energy Allocation System (EAS) generates a set of falsifiable predictions regarding the relationship between endocrine signaling patterns and bioenergetic capacity under controlled conditions. Representative hypotheses include:

**H1.**  *Under experimentally induced metabolic stress, reductions in the free T3:T4 ratio will correlate more strongly with direct or functional measures of skeletal muscle mitochondrial capacity (e.g., reserve respiratory capacity, exercise recovery kinetics, or lactate clearance) than with circulating TSH concentrations.*

**H2.**  *Individuals with preserved mitochondrial reserve capacity will demonstrate coordinated, transient activation of the HPA axis with rapid post-stress recovery, whereas individuals with constrained bioenergetic reserve will exhibit prolonged or dysregulated HPA signaling independent of equivalent stress exposure.*

**H3.**  *Suppression of gonadal axis signaling during sustained stress will be proportional to markers of energetic constraint (e.g., inflammatory load or impaired metabolic flexibility) rather than to stress exposure alone.*

**H4.**  *Restoration of bioenergetic reserve through interventions that reduce energetic demand or improve recovery capacity will precede and predict normalization of coordinated endocrine signaling patterns across the HPA, HPT, and HPG axes, rather than isolated correction of individual hormone levels.*

Failure to observe these predicted relationships would argue against the EAS framework, whereas their confirmation would support the model’s central premise that endocrine coordination reflects underlying energetic constraints rather than isolated glandular dysfunction.

Perceived energy is not solely determined by physiological load but is modulated by effort–reward valuation. Experimental evidence indicates that task completion under favorable reward conditions can transiently increase perceived energy, whereas sustained effort with inadequate reward depletes it [62]. Within the EAS framework, this suggests that cognitive appraisal dynamically influences energy allocation decisions rather than merely reflecting downstream fatigue.

The EAS framework generates several testable implications that distinguish energetic configurations without requiring novel biomarkers. Because these states reflect coordinated system-level behavior, we propose that they are best assessed through patterns rather than isolated measurements. Endocrine profiles, inflammatory markers, metabolic indices, and symptom clusters are expected to co-vary in ways that reflect underlying energetic availability rather than primary glandular dysfunction [7,34].

For example, mobilization-dominant configurations are predicted to associate with elevated or dysregulated HPA activity, altered thyroid hormone conversion ratios, and early suppression of gonadal signaling, whereas conservation-dominant states are expected to show broader suppression of anabolic and metabolic throughput alongside immune tolerance markers. Longitudinal assessment should reveal that transitions between configurations track changes in energetic demand and recovery capacity rather than static diagnoses.

In addition to biochemical and physiological measures, the Energy Allocation System predicts that subjective and functional readouts provide meaningful information about the underlying energetic state. Stress perception, sleep quality, fatigue, and recovery capacity are not epiphenomena but reflect the organism’s integrated response to energetic availability and demand across neuroendocrine and metabolic systems. Validated psychometric instruments capture behavioral expressions of energetic allocation. Measures of sleep quality, perceived stress, and resilience reflect circadian alignment, anticipatory HPA mobilization, and recovery efficiency, respectively, rather than isolated psychological states [63,64,65]. Within the EAS framework, these tools function not as diagnostic tools but rather as practical proxies for coordinated system-level energetic behavior when direct assessment of bioenergetic reserve is impractical.

Instruments such as the 36-item Thyroid Symptom Questionnaire (TSQ-36), Pittsburgh Sleep Quality Index (PSQI), Perceived Stress Scale (PSS), and Brief Resilience Scale (BRS) capture distinct but interrelated dimensions of metabolic pacing, recovery efficiency, stress appraisal, and adaptive flexibility [59,66,67,68]. Patterns across multiple instruments are therefore expected to align with energetic configurations and to change dynamically as energetic demand and recovery capacity shift. Representative psychometric and functional readouts mapped to energetic dimensions are summarized in Appendix A Table A3.

Importantly, the EAS predicts that interventions restoring bioenergetic reserve, whether through reduction in inflammatory load, improvement of metabolic flexibility, circadian alignment, or stress recovery, will normalize endocrine signaling patterns more effectively than isolated hormone modulation alone. These predictions provide a framework for hypothesis-driven observational studies and interventional trials examining resilience, recovery, and multisystem dysfunction.

### 5.5. Proposed Energetic Phenotypes (Heuristic Clusters)

The Energy Allocation System (EAS) further predicts that recurrent patterns of endocrine signaling, immune behavior, and metabolic throughput can be grouped into heuristic energetic phenotypes. To enhance interpretability while avoiding diagnostic overreach, energetic phenotypes are described using proposed directional trends in representative biomarkers (e.g., ↑/↓), rather than absolute thresholds. These directional patterns are intended as operational heuristics reflecting coordinated system-level behavior under energetic constraint, not as criteria for disease classification. Accordingly, these phenotypes are not intended as diagnostic categories but as conceptual clusters that reflect how energy is preferentially allocated under differing conditions of demand, reserve capacity, and recovery efficiency. Their purpose is to operationalize the EAS framework for hypothesis generation, observational research, and pattern recognition, rather than to replace disease-based classification systems [1]. Representative heuristic phenotypes and associated system-level patterns are summarized in Appendix A Table A1 and Table A2.

Across the experimental and clinical literature, stress exposure, inflammation, circadian disruption, and metabolic inflexibility consistently co-vary with coordinated alterations across the HPA, HPT, and HPG axes [2,7]. Within the EAS, these coordinated responses are interpreted as system-level adaptations to energetic constraint, producing recognizable configurations that persist as long as underlying energetic conditions remain unchanged.

#### 5.5.1. Mobilization-Biased Phenotype

This phenotype reflects prioritization of rapid energy mobilization to meet sustained or recurrent demands. It is characterized by heightened or prolonged HPA engagement, constrained thyroid-mediated metabolic throughput, and early suppression of gonadal investment. Immune activity in this state tends toward energetically efficient, inflammation-prone signaling, while recovery processes are deferred. Although function may be preserved in the short term, prolonged residence in this phenotype is associated with accumulating energetic cost and reduced resilience capacity [2,3].

#### 5.5.2. Throughput-Constrained Phenotype

In this configuration, thyroid-mediated metabolic pacing becomes a primary limiting factor despite continued demand. Altered thyroid hormone signaling and peripheral conversion patterns reduce mitochondrial throughput, constraining ATP availability across systems. HPA activation may remain present but less effective, while HPG signaling is suppressed to conserve energy. This phenotype is commonly observed in contexts of chronic stress, illness, or inflammation where energetic supply cannot meet demand despite ongoing mobilization efforts [9,45].

#### 5.5.3. Conservation-Dominant Phenotype

Under conditions of sustained energetic depletion or impaired recovery, the EAS predicts a shift toward broad energy conservation. This phenotype is marked by reduced metabolic throughput, suppression of anabolic and reproductive investment, and immune tolerance-oriented behavior. Endocrine signaling patterns in this state favor minimization of energetic expenditure and protection against further depletion rather than performance optimization. While adaptive in the short term, prolonged persistence limits functional recovery and adaptive flexibility [7,36,51,69].

#### 5.5.4. Resilient Allocation Phenotype

In contrast, individuals with preserved mitochondrial reserve capacity and efficient recovery demonstrate coordinated endocrine engagement without dominance of a single axis. HPA activation remains transient and proportional, thyroid signaling supports flexible metabolic pacing, and gonadal investment is maintained. Immune responses remain effective without excessive energetic cost. This phenotype reflects energetic sufficiency, enabling rapid transitions between mobilization, repair, and long-term investment states [5,27,55].

#### 5.5.5. Utility and Scope of Phenotypic Classification

These heuristic phenotypes are intended to support pattern-based interpretation of endocrine, metabolic, immune, and symptomatic data rather than isolated biomarker thresholds. Their value lies in identifying dominant energetic strategies and predicting how individuals may respond to stress exposure, recovery interventions, or therapeutic modulation. Importantly, transitions between phenotypes are expected to occur as energetic conditions change, underscoring the dynamic and reversible nature of energy allocation states. An illustrative example of how energetic configurations predicted by the Energy Allocation System may be evaluated experimentally is provided in Box 1.

Box 1 **Example Experimental Test of the Energy Allocation System (EAS): A Hypothesis-Generating Study Design.**

    The Energy Allocation System (EAS) framework generates testable predictions regarding how coordinated endocrine and immunometabolic patterns shift in response to changes in energetic demand and recovery capacity. One illustrative approach to evaluating these predictions is a longitudinal, hypothesis-generating intervention study designed to assess transitions between energetic configurations rather than isolated biomarker changes.

***Study* *Population***

    Adults experiencing chronic psychosocial or physiological stress but without overt endocrine disease would be recruited. Participants may be stratified at baseline using heuristic energetic phenotypes (Appendix A Table A1) derived from coordinated endocrine, metabolic, and functional readouts.

***Intervention***

    A multimodal intervention aimed at restoring bioenergetic reserve rather than directly modulating individual hormones. Components may include circadian alignment strategies, reduction in inflammatory load, improvement of metabolic flexibility, and structured stress-recovery practices. The intervention is selected to reduce energetic demand and enhance recovery capacity simultaneously.

***Outcome* *Domains***

    Primary outcomes focus on pattern-level changes rather than single analytes, including:

- Coordination across HPA, HPT, and HPG axes;
- Shifts in inflammatory tone and metabolic flexibility;
- Improvements in recovery efficiency and functional resilience.

    Secondary outcomes include psychometric and functional measures reflecting stress perception, sleep quality, fatigue, and resilience capacity (Appendix A Table A3).

***Predicted* *Findings***

    The EAS predicts that effective restoration of energetic reserve will be associated with:

- Reduced dominance of mobilization-biased or conservation-dominant configurations;
- Improved endocrine coordination across axes;
- Enhanced recovery capacity and adaptive flexibility.

    Importantly, these improvements are expected to emerge through system-level reorganization rather than normalization of any single biomarker.

***Interpretive* *Value***

    Such a study would allow evaluation of whether energetic configurations are dynamic and reversible, and whether changes in bioenergetic context precede or accompany coordinated endocrine normalization. This approach emphasizes falsifiability of the EAS framework while avoiding assumptions of linear causality or single-axis primacy.

## 6. Discussion

This review advances the Energy Allocation System (EAS) as a unifying framework linking mitochondrial bioenergetics with coordinated endocrine regulation to explain resilience and vulnerability under stress. By foregrounding energy availability as the primary constraint on adaptive capacity, the EAS integrates established findings across stress biology, endocrinology, immunometabolism, and mitochondrial physiology into a coherent explanatory model. Rather than proposing new pathways, this framework reframes well-characterized mechanisms through an energetic lens that clarifies why multisystem alterations frequently co-occur in clinical and experimental settings. An example hypothesis-generating study design illustrating how energetic configurations may be evaluated longitudinally is outlined in Box 1.

### 6.1. Relationship to Existing Models of Stress and Adaptation

The EAS complements and extends existing models of stress adaptation, particularly the concepts of allostasis and allostatic load [2]. While allostatic load describes the cumulative cost of repeated physiological adjustments to stress, it does not specify the mechanistic substrate through which these costs accumulate. The EAS addresses this gap by identifying mitochondrial reserve capacity and bioenergetic throughput as the limiting resources that mediate adaptive trade-offs. In this view, allostatic burden reflects not only signal intensity or duration but also the energetic context in which adaptive responses occur.

Similarly, the EAS aligns with models of metabolic flexibility and adaptive homeostasis by emphasizing the organism’s capacity to adjust energy utilization in response to changing demands. However, by explicitly integrating endocrine axes as regulators of energy allocation across timescales, the EAS provides a structured explanation for coordinated changes in stress responsiveness, metabolic pacing, immune tolerance, and reproductive investment. This integration helps reconcile observations that are otherwise treated as parallel or unrelated phenomena.

### 6.2. Explaining Multisystem Co-Occurrence and Clinical Heterogeneity

A recurring challenge in both research and clinical practice is the frequent co-occurrence of endocrine alterations, immune dysregulation, metabolic inflexibility, and neurocognitive symptoms in the absence of overt pathology. The EAS offers a parsimonious explanation for this clustering by framing these changes as coordinated responses to energetic constraint rather than independent system failures. Variability in mitochondrial reserve capacity, inflammatory load, and recovery efficiency can produce divergent physiological patterns even under similar stress exposures, accounting for heterogeneity in clinical presentations.

This perspective also clarifies why interventions targeting single hormones or pathways often yield incomplete or transient benefits. When energetic constraints persist, downstream endocrine modulation may temporarily alter signaling without restoring the underlying capacity for recovery. Conversely, interventions that reduce energetic load or enhance bioenergetic reserve, whether through improved metabolic flexibility, inflammation reduction, circadian alignment, or stress recovery, may normalize endocrine patterns indirectly by relieving constraint rather than forcing output.

Mood and behavioral phenotypes provide clinically observable expressions of energetic allocation. Across psychiatric and non-psychiatric populations, stress exposure is associated with coordinated alterations in thyroid hormone signaling, insulin sensitivity, and inflammatory tone, even in the absence of overt endocrine disease. These findings support the interpretation of depressive or hypoactive states as thyroid-mediated energy conservation configurations, whereas hyperactive or manic states reflect sustained mobilization with elevated metabolic throughput [70,71,72]. Observations from primary mitochondrial disorders and states of nutritional or energetic insufficiency provide additional biological precedents and further support the plausibility of this framework. Individuals with inherited mitochondrial disease exhibit reduced stress tolerance, multisystem vulnerability, and exaggerated physiological consequences in response to otherwise modest energetic demands, despite heterogeneous genetic etiologies [73,74]. Similarly, nutritional deprivation, chronic inflammation, or illness-associated energy deficit is associated with coordinated endocrine suppression, immune reconfiguration, and impaired recovery capacity [75,76,77]. While these conditions are not equivalent to the adaptive states described here, they illustrate how diminished bioenergetic reserve can constrain system-level resilience and shape endocrine and immune behavior across tissues.

Similar energetic trade-offs are observed in neuronal systems. Acute stress exposure is associated with sympathetic and HPA-mediated mobilization that enhances arousal, attention, and threat detection, supporting short-term cognitive performance. Under sustained energetic demand, however, prolonged neuroendocrine activation and reduced metabolic reserve are associated with shifts toward hypervigilance, sleep disruption, and impaired cognitive flexibility. In contrast, conservation-dominant energetic states are frequently characterized by reduced neuronal metabolic throughput, manifesting as cognitive slowing, fatigue, and diminished executive function [78,79,80]. As with immune cells, these patterns are not interpreted as intrinsic neuronal dysfunction but as context-dependent reallocations of energetic support shaped by endocrine prioritization and bioenergetic constraint.

### 6.3. Adaptive Versus Maladaptive Persistence

An important implication of the EAS framework is the distinction between adaptive reallocation and maladaptive persistence. Suppression of thyroid or gonadal signaling, immune tolerance shifts, and reliance on HPA-mediated mobilization are not inherently pathological; they represent conserved strategies for energy conservation under strain. However, when stressors persist without sufficient recovery, these adaptive configurations may become entrenched, narrowing the organism’s adaptive range and reducing resilience capacity over time.

This distinction cautions against interpreting energetic conservation states as primary dysfunctions requiring direct correction. Instead, it emphasizes the importance of identifying and addressing the upstream energetic conditions that maintain these configurations. Within this framework, resilience decline reflects not failure of adaptation but prolonged residence in protective states necessitated by unresolved energetic imbalance.

### 6.4. Limitations and Scope of the Framework

In addition to contemporary stress exposure, energetic allocation capacity is further shaped by early life experiences through epigenetic programming. Adverse early exposures induce lasting methylation changes in genes regulating glucocorticoid signaling, thyroid axis responsiveness, immune modulation, and reproductive investment, including *NR3C1* and *TRH* promoter regions [81,82,83]. These modifications alter endocrine set points and stress responsivity across the lifespan, providing a mechanistic basis for persistent interindividual differences in resilience capacity that cannot be explained by current stress exposure alone.

Several limitations of the EAS should be acknowledged. First, this model is conceptual and integrative, drawing on existing empirical literature rather than presenting novel experimental data. While it generates testable predictions, validation will require longitudinal and interventional studies explicitly designed to assess energetic reserve, endocrine coordination, and recovery dynamics simultaneously. Consistent with its framing in the Introduction, the EAS is intended as a heuristic organizational framework that integrates established biological mechanisms, rather than as a replacement for existing disease models or a claim of mechanistic novelty.

A related limitation is the potential for circular interpretation if energetic constraint is inferred solely from the coordinated endocrine and metabolic patterns that the EAS seeks to interpret. To mitigate this risk, the framework is not intended to operate as a closed explanatory loop. Instead, energetic constraint must be anchored to independent indicators of energetic demand, reserve, and recovery, including longitudinal stress exposure, functional performance, recovery kinetics, or convergent physiological signals across systems. Emphasis is therefore placed on pattern coherence and temporal dynamics rather than attribution based on any single biomarker or axis.

Importantly, the EAS does not assume that energetic constraint is always the initiating driver of endocrine dysregulation. Primary endocrine pathologies—such as intrinsic thyroid, adrenal, or gonadal disease—may precede and impose energetic constraints rather than arise from them. Within the EAS, such conditions are conceptualized as upstream perturbations that alter energy allocation dynamics, potentially accelerating or exaggerating constraint-associated patterns across other physiological systems. This bidirectional framing allows the model to accommodate both primary endocrine dysfunction and secondary adaptive responses without presuming causal directionality.

The EAS does not claim that energy allocation alone explains all aspects of resilience. Psychological, social, behavioral, and environmental factors exert powerful influences on stress perception, coping strategies, and recovery opportunities. Rather, the framework proposes that energetic constraints form the biological substrate upon which these factors exert their effects. Individual variability in genetics, developmental programming, and environmental exposures will shape how these principles manifest, even as the underlying architecture remains conserved. Accordingly, the EAS is intended to complement psychosocial and behavioral models of resilience by specifying the energetic substrate through which these influences exert physiological effects.

Finally, measurement of bioenergetic capacity at the organismal level remains indirect. While mitochondrial reserve capacity can be assessed experimentally in isolated systems, practical clinical proxies require further refinement. This limitation underscores the need for integrated assessment strategies that emphasize patterns and trajectories rather than isolated biomarkers.

### 6.5. Implications for Future Research

The EAS provides a foundation for hypothesis-driven research examining resilience as a dynamic, energy-constrained process. Future studies may test whether transitions between energetic configurations predict functional outcomes more accurately than static endocrine measures, and whether interventions that restore energetic reserve facilitate coordinated normalization across endocrine axes. Longitudinal designs capturing stress exposure, recovery, and energetic status will be particularly valuable in evaluating the reversibility and stability of the configurations described here.

## 7. Conclusions

This review presents the Energy Allocation System (EAS) as an integrative framework for understanding resilience as a function of bioenergetic constraint and coordinated endocrine regulation. By positioning mitochondrial reserve capacity as the limiting substrate for adaptive responses, the EAS clarifies how the hypothalamic-pituitary-adrenal, thyroid, and gonadal axes operate together to prioritize energy use across acute mobilization, metabolic pacing, and long-term investment. Within this framework, endocrine alterations commonly observed under stress are reframed as context-dependent reallocations rather than isolated dysfunctions.

The EAS provides a mechanistic explanation for the frequent co-occurrence of endocrine, immune, metabolic, and neurocognitive changes in both experimental and clinical settings. It accounts for variability in stress tolerance and recovery by emphasizing energetic availability and system-wide coordination, offering a parsimonious lens through which heterogeneous presentations can be understood without invoking multiple independent pathologies. Importantly, this framework distinguishes adaptive conservation from maladaptive persistence, underscoring the role of unresolved energetic strain in the erosion of resilience over time.

Taken together, the Energy Allocation System provides a parsimonious heuristic framework for integrating endocrine, immune, and metabolic patterns under energetic constraint, with the explicit recognition that empirical validation and refinement are required.

By synthesizing established principles across bioenergetics, endocrinology, and immunometabolism, the EAS generates testable predictions regarding energetic configurations, recovery dynamics, and intervention responsiveness. This model offers a structured foundation for future research and clinical inquiry focused on resilience as a dynamic, energy-constrained process. Framing adaptation through energy allocation may help unify disparate observations and guide more effective strategies for restoring systemic resilience under conditions of chronic stress. Viewed together, these considerations position energy allocation as a unifying constraint linking stress exposure, endocrine coordination, and resilience capacity.

## Data Availability

No new data were created or analyzed in this study.

## Abbreviations

The following abbreviations are used in this manuscript:

HPA	Hypothalamic-Pituitary-Adrenal
ATP	Adenosine Triphosphate
HPT	Hypothalamic-Pituitary-Thyroid
HPG	Hypothalamic-Pituitary-Gonadal
EAS	Energy Allocation System
Nrf2	Nuclear factor erythroid 2-related factor 2
IL-6	Interleukin-6
CRP	C-reactive protein
TNF-α	Tumor necrosis factor-α
GnRH	Gonadotropin-releasing hormone
LH	Luteinizing hormone
FSH	Follicle-stimulating hormone
SNS	Sympathetic nervous system
T4	Thyroxine
T3	Triiodothyronine
rT3	Reverse T3
TSH	Thyroid-stimulating hormone
CRH	Corticotropin-releasing hormone
Treg	Regulatory T-cell
CAR	Cortisol awakening response
TSQ-36	Thyroid Symptom Questionnaire
PSQI	Pittsburgh Sleep Quality Index
PSS	Perceived Stress Scale
BRS	Brief Resilience Scale

## Appendix A

**Table A1:** Proposed Energetic Phenotypes within the Energy Allocation System (EAS).

Energetic Phenotype	Dominant Allocation Strategy	Endocrine Pattern (Directional)	Bioenergetic Characteristics	Representative Physiological/Clinical Correlates *	Temporal Characteristics/Reversibility
Resilient Allocation	Balanced distribution across systems	HPA: ↔ appropriate diurnal rhythm and stress reactivity Thyroid: ↔ fT3; ↔ rT3/fT3 ratio HPG: ↔ sex steroids and gonadotropins (contextual)	Adequate mitochondrial reserve capacity; efficient substrate switching; preserved recovery capacity	Flexible stress responsiveness; stable energy levels; preserved immune competence; efficient recovery following stress	Dynamic and rapidly reversible; maintained when energetic reserve and recovery capacity are sufficient
Mobilization-Biased	Prioritization of rapid energy mobilization	HPA: ↑ cortisol output, variability, or prolonged activation Thyroid: ↓ fT3; ↑ rT3/fT3 ratio HPG: ↓ sex steroids (relative to context)	Increased reliance on glucocorticoid-mediated substrate mobilization; rising energetic cost	Sustained alertness under demand; reduced recovery capacity; early fatigue; heightened inflammatory tone	Typically adaptive in the short term; risk of persistence increases with unresolved stress or impaired recovery
Throughput-Constrained	Limited metabolic pacing despite ongoing demand	Thyroid: ↓ fT3; ↑ T4:fT3 ratio; ↑ rT3/fT3 ratio HPA: ↔ or ↓ responsiveness relative to demand HPG: ↓ signaling under sustained strain	Reduced mitochondrial throughput; impaired ATP availability relative to demand	Low stamina; cold intolerance; cognitive slowing; metabolic inflexibility under stress	May emerge gradually under sustained strain; generally reversible with restoration of energetic reserve and metabolic flexibility
Conservation-Dominant	Broad energy conservation and tolerance	Thyroid: ↓ fT3; ↓ thyroid-mediated metabolic activity HPG: ↓ sex steroids and gonadotropins HPA: ↔ blunted or flattened output	Low mitochondrial reserve capacity; prioritization of survival over performance	Fatigue; impaired recovery; immune tolerance-oriented responses; reduced anabolic capacity	Protective under acute or severe strain; persistence reflects unresolved energetic constraint rather than fixed pathology
Recovery-Limited/Persistent Conservation †	Entrenched conservation under unresolved strain	Flattened or dysregulated endocrine signaling across axes	Chronically depleted energetic reserve; narrow adaptive range	Reduced resilience; poor response to stressors or interventions; prolonged symptom persistence	Represents prolonged residence in a conservation-dominant state; reversibility depends on successful restoration of energetic capacity

Notes: * Representative correlates are descriptive and intended to illustrate system-level patterns rather than diagnostic criteria. Directional arrows (↑/↓/↔) denote relative trends rather than absolute thresholds and are used for heuristic pattern recognition. † The “Recovery-Limited/Persistent Conservation” phenotype reflects prolonged residence in a conservation-dominant allocation strategy rather than a distinct pathological entity.

**Table A2:** Representative Biomarker Domains and Functional Readouts Associated with Energetic Phenotypes.

Domain	Representative Measures	Pattern Consistent with Energetic Constraint	Interpretive Value within EAS *
HPA Axis Activity	Diurnal cortisol rhythm; cortisol awakening response; stress reactivity	↑ or flattened diurnal rhythm; impaired recovery	Reflects prioritization of rapid mobilization and energetic cost of stress adaptation
Thyroid Axis Signaling	TSH, free T4, free T3; T4:T3 ratio; rT3:fT3 ratio	↓ fT3; ↑ rT3/fT3; ↑ T4:fT3	Indicates capacity for mitochondrial stimulation and metabolic pacing under demand
Gonadal Axis Activity	Sex steroid levels; gonadotropins	↓ sex steroids and/or gonadotropins	Serves as a proxy for long-term energetic investment and tolerance capacity
Inflammatory Tone	CRP; cytokine profiles; immune cell polarization markers	↑ low-grade inflammation or tolerance-oriented signaling	Reflects energetic cost of immune activity and trade-offs with other systems
Metabolic Flexibility	Glucose variability; lipid utilization patterns; insulin sensitivity indices	↓ substrate switching efficiency; ↑ reliance on stress-mediated substrates	Indicates efficiency of energy utilization under fluctuating demand
Mitochondrial Function (Indirect)	Micronutrient sufficiency (iron, selenium, iodine, folate, cholecalciferol, cobalamin); lactate dynamics; exercise tolerance; recovery kinetics	↓ tolerance and recovery; ↑ exertional fatigue	Serves as a functional proxy for bioenergetic reserve at the organismal level
Methylation Status (Functional)	SAM:SAH ratio; homocysteine; folate-dependent one-carbon intermediates; epigenetic markers where available	↓ methylation capacity; ↑ homocysteine; altered SAM:SAH ratio	Reflects longer-timescale regulation of stress adaptation and modulates persistence of energy allocation states rather than acute configuration
Circadian Integrity	Sleep–wake timing; actigraphy; melatonin or cortisol rhythms	↓ amplitude; phase disruption	Modulates efficiency of energy allocation and recovery processes
Neurocognitive Performance	Attention, processing speed, working memory, cognitive endurance	↓ cognitive stamina; ↑ mental fatigue under sustained demand	Reflects energetic availability for high-cost neural functions
Symptom & Functional Indices	Fatigue burden, exertional tolerance, sleep quality, recovery perception	↑ symptom burden; ↓ functional tolerance and recovery efficiency	Captures integrated system-level output of energetic allocation

Notes: * Biomarkers and functional measures are intended to be interpreted as coordinated patterns rather than isolated diagnostic indicators. No single measure is sufficient to assign an energetic phenotype, and directional trends should be interpreted in context. Directional arrows (↑/↓) denote relative trends rather than absolute thresholds and are used for heuristic pattern recognition.

**Table A3:** Psychometric and Functional Questionnaires as System-Level Readouts within the Energy Allocation System (EAS).

Instrument	Primary Domain Assessed	Energetic Dimension Reflected	Pattern Consistent with Energetic Sufficiency	Pattern Consistent with Energetic Constraint	Interpretive Role within EAS *
TSQ-36 (Thyroid Symptom Questionnaire)	Multisystem symptom burden related to thyroid-regulated processes	Metabolic pacing and energetic throughput	Minimal or fluctuating symptom burden proportional to demand	Persistent multisystem symptoms reflecting constrained metabolic throughput	Serves as a functional proxy for thyroid-mediated energy distribution rather than isolated glandular dysfunction
PSQI (Pittsburgh Sleep Quality Index)	Sleep quality and circadian integrity	Recovery efficiency and circadian coordination	Consolidated sleep with effective overnight recovery	Fragmented or non-restorative sleep patterns	Reflects adequacy of nocturnal recovery and efficiency of energy reallocation
PSS (Perceived Stress Scale)	Subjective stress appraisal	HPA engagement relative to energetic reserve	Stress perceived as manageable and proportionate to context	Persistent perceived stress disproportionate to situational demand	Captures interaction between energetic constraint and cognitive–emotional appraisal
BRS (Brief Resilience Scale)	Capacity to recover from stress	Global resilience and adaptive flexibility	Rapid return to baseline following stressors	Prolonged recovery and difficulty returning to baseline	Integrates energetic availability with system-level recovery capacity
Fatigue Severity Scales †	Physical and cognitive fatigue	Energetic cost and reserve depletion	Fatigue resolves with rest and recovery	Persistent fatigue despite rest	Reflects cumulative energetic burden across systems
Cognitive Performance Tasks †	Attention, working memory, processing speed	Neural energetic availability	Stable cognitive performance under load	Cognitive slowing or mental fatigue	Indicates allocation of energy to high-cost neural functions

Notes: * Questionnaires and functional measures are interpreted as readouts of coordinated system-level behavior rather than diagnostic instruments. Patterns across multiple measures provide greater interpretive value than any single score. † Examples include validated fatigue scales or cognitive task batteries commonly used in stress and resilience research; specific instruments may be selected based on study or use context.
